# Urea-driven g-C_3_N_4_ nanostructures for highly efficient photoreduction of Cr(vi) under visible LED light: effects of calcination temperature

**DOI:** 10.1039/d4ra00859f

**Published:** 2024-08-27

**Authors:** Faramarz Safari, Reza Poursalehi, Hamid Delavari

**Affiliations:** a Nanotechnology Group, Faculty of Engineering and Technology, Tarbiat Modares University Tehran Iran poursalehi@modares.ac.ir

## Abstract

Graphitic carbon nitride (g-C_3_N_4_) nanostructures were synthesized *via* the calcination of urea at various temperatures ranging between 400 and 600 °C and were utilized for photoreduction of Cr(vi) in aqueous medium. Due to the low adsorption of Cr(vi) on the g-C_3_N_4_ surface, a more accurate assessment of the photocatalytic performance of the samples was carried out. Although the characterization showed that the specific surface of samples increased as the calcination temperature increased, the most efficient product in terms of the photoreduction duration of Cr(vi) was produced through the calcination process carried out at 450 °C, which reduced the concentration by more than 99% in 40 minutes. These results demonstrate that the structural and surface properties of g-C_3_N_4_ are critical factors that impact the photocatalytic performance. Alongside the calcination temperatures, the concentration of citric acid as a hole scavenger, the source of illumination, pH levels, and the recycling ability of the produced specimen at 450 °C were also investigated. Conspicuously, the photocatalyst works better when more citric acid is present and the pH level decreases. Out of all the cases studied regarding the light source, the 400 nm LED light source was found to be the most efficient. Additionally, even after going through the photoreduction process four times, the photocatalyst still remained highly efficient.

## Abstract 2

The effectiveness of g-C_3_N_4_ samples, obtained at different calcination temperatures, was examined in the photoreduction of Cr(vi). Band positions and the Cr(vi) photoreduction mechanism of g-C_3_N_4_ are also reported.

## Graphical abstract

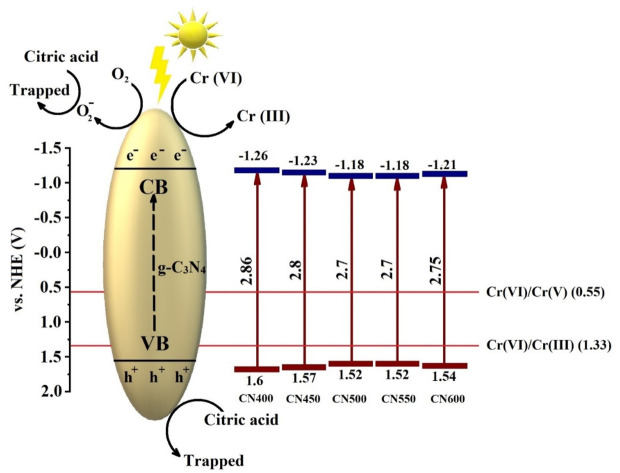

## 1. Introduction

Hexavalent chromium (Cr(vi)), a hypertoxic heavy metal ion, is prevalent in wastewater originating from various industries such as electroplating, pigment coloring, petroleum refining, and leather tanning.^1,2^ Cr(vi) has been categorized as a potent carcinogen and mutagen with a significant impact on the health of both humans and animals. The World Health Organization has identified Cr(vi) as a matter of paramount concern with regard to toxic pollutants. With respect to drinking water standards, the recommended maximum acceptable concentration of Cr(vi) is below 50 μg L^−1^.^3,4^ Numerous methodologies, inclusive of adsorption, ion exchange, membrane separation, chemical precipitation, bioremediation, and photocatalytic reduction, have been created to eliminate Cr(vi) from wastewater or facilitate its detoxification to the comparatively less hazardous Cr(iii).^5,6^ However, it is important to note that every technique has its own inherent limitations. For instance, adsorption may suffer from saturation, ion exchange may generate sludge and the photoreduction process may require photocatalyst separation. Hence, giving due consideration to the prevailing circumstances and selecting and refining the most fitting approach is a pivotal responsibility that cannot be disregarded.^7,8^

**Fig. 1:**
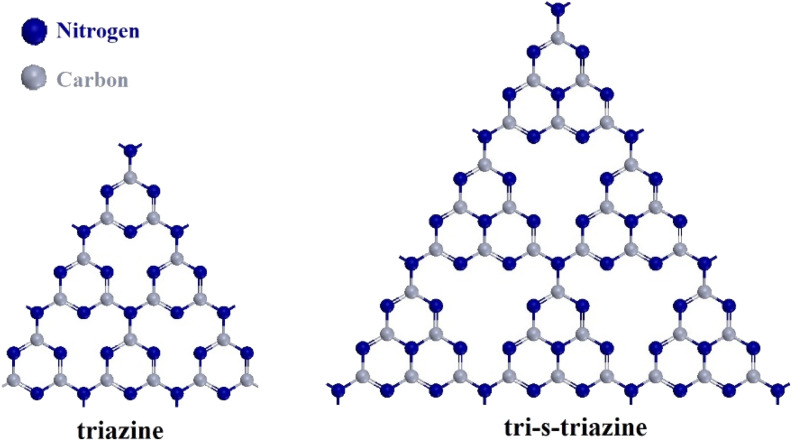
Two fundamental structures of g-C_3_N_4_.

Extensive research has been conducted on renewable, cost-efficient, eco-friendly and highly efficient photocatalytic reactions for the purpose of reducing organic pollutants or heavy metals.^9,10^ In the past few decades, several materials including TiO_2_, ZnO, WO_3_, CdS, Bi_2_S_3_ and Fe_2_O_3_ nanostructures have been reported to exhibit photocatalytic properties.^11–15^ Over the past decade, significant interest has been devoted to graphitic carbon nitride (g-C_3_N_4_), which is a metal-free polymeric two dimensional (2D) semiconductor that has a narrow band gap that enables effective visible light absorption.^16^ g-C_3_N_4_ is a π-conjugated polymeric n-type semiconductor, with its two chemical structures depicted in Fig. 1.^17,18^ Moreover, the distinctive characteristics of g-C_3_N_4_, such as its low cost and straightforward synthesis, the potential for adjustability and resistance to thermal and chemical degradation, have garnered significant attentions as a research target within the realm of photocatalysis.^19^ Numerous investigations have additionally directed attention towards the amalgamation and arrangement of g-C_3_N_4_ with other semiconductors to refine properties and conduct band gap engineering, as evidenced by a range of studies.^20–22^

Various organic precursors, including cyanamide, dicyanamide, melamine, urea and thiourea, have been employed in the synthesis of g-C_3_N_4_ through polymerization reactions.^23^ Calcination at atmospheric pressure is a commonly utilized method that is fairly easy to perform in a furnace. The properties of the resultant product are greatly influenced by the conditions under which the heating is conducted, such as the heating rate from room temperature, along with the duration and temperature of the calcination process. Various photoreactions were utilized to investigate the property disparities of g-C_3_N_4_, which was synthesized employing different precursors under diverse conditions. The investigation of calcination temperature has been a prevalent parameter for various chemical reactions, including CO_2_ conversion, H_2_ production, NO oxidation and dyes photodegradation, such as those observed in methylene blue, methyl orange, rhodamine B and murexide.^24–30^ Moreover, analogous photoreactions have examined the impact of various factors on the outcome of the reaction, including the precursor substance, heating rate, and duration of calcination.^31–33^

Investigation of the g-C_3_N_4_ efficiency with Cr(vi) photoreduction, besides having environmental benefits, can help to better understand the photoactivity of g-C_3_N_4_ because of the very insignificant adsorption of Cr(vi) on the g-C_3_N_4_ surface. Based on the results of studies on the effects of calcination temperature, the specific area of g-C_3_N_4_ increases as the calcination temperature increases.^25,26^ The establishment of the adsorption–desorption equilibrium is a common step before the irradiation of the light source in photoreaction experiments and it has also an effect on the reaction kinetics.^34^ In addition, due to the adsorption of dyes on the g-C_3_N_4_ surface, the dye removal efficiency in the adsorption–desorption equilibrium step has been increased by surface area increase. This issue causes different mass transfer conditions at the irradiation step beginning including concentration discrepancy in solution and different concentration gradian in the binary layer on the photocatalyst surface.^35^ As well as dye adsorption on the photocatalyst surface can affect light adsorption.^36^ The effect of precursors on g-C_3_N_4_ in the photoreduction of Cr(vi) has been studied by Liang *et al.* and urea has performed the best efficiency between dicyandiamide, melamine, thiourea, and urea.^37^ Furthermore, numerous studies have been conducted on the improvement of g-C_3_N_4_ properties by adding various material for better photoreduction of Cr(vi).^38,39^ Yang *et al.* studied the composition of g-C_3_N_4_ by three metal oxides, including TiO_2_, Fe_3_O_4_@SiO_2_ by hydro-gel synthesis method which the best performance was seen by the sample that calcinated at 450 °C.^40^ Wang *et al.* studied the g-C_3_N_4_/ZnS coupled composite synthesized at 600 °C that can reduce more than 90% of Cr(vi) solution with a concentration of 10 mg L^−1^ in 120 min under 500 W xenon lamp.^41^ Xiao *et al.* succeeded in photoreduction of Cr(vi) by adding α-Fe_2_O_3_ to g-C_3_N_4_ by hydrothermal method.^42^ By doping Br in the g-C_3_N_4_ structure, Wang *et al.* reduced Cr(vi) with a concentration of 20 mg L^−1^ by 60%.^43^ Also, studies have been conducted on the synthesis of MoS_2_ and WO_3_ heterostructures with g-C_3_N_4_, and these materials have improved the performance of g-C_3_N_4_ in removing the Cr(vi).^44,45^ Adding other semiconductors, including metal oxides, can be a useful solution for band gap engineering as a result of improving photocatalytic performance. On the other hand, the release of these compounds amid the reaction can cause harmfulness and be challenging. Hence, engineering the properties of carbon nitride alone as a metal-free semiconductor can be a great arrangement for the photoreduction of Cr(vi).

Within the present study, the synthesis of g-C_3_N_4_ was conducted through the calcination of urea at varying temperatures ranging from 400 to 600 °C for the investigation of Cr(vi) photoreduction performance. The products were subsequently characterized, including their structural, morphological, optical, and photoreduction performance in regard to Cr(vi). To the best of our knowledge, investigating the impact of urea calcination temperature on the performance of g-C_3_N_4_ for the photoreduction of Cr(vi) has not been reported so far. The photocatalyst synthesized at 450 °C exhibited superior efficacy in photoreduction, leading to its selection for further assessment with respect to recycling capacity, citric acid level, light source, and pH impacts.

## 2. Materials and methods

### 2.1. Chemicals

Urea (Fluka), potassium dichromate (Ghatran Shimi), citric acid, and 1,5-diphenylcarbazide (Merck), acetone, phosphoric acid, sulfuric acid and sodium hydroxide (Neutron) are of analytical grade and used without further purification.

### 2.2. Preparation of g-C_3_N_4_

g-C_3_N_4_ was synthesized through the thermal polymerization of urea at various temperatures. Typically, 30 g of urea powder was introduced into a covered alumina crucible along with a volume of 50 mL, after which it was subjected to a heating process at a rate of 5 °C min^−1^ until reaching temperatures of 400, 450, 500, 550, and 600 °C over a period of 4 hours within a furnace. Upon cooling the furnace to ambient temperature, g-C_3_N_4_ powders were collected and were named respectively CN400, CN450, CN500, CN550 and CN600 according to the temperature of the polymerization reaction.

### 2.3. Characterization

The X-ray diffraction (XRD) patterns of the powders were provided by a PANalytical X'Pert Pro instrument with Cu-Kα radiation (*k* = 1.5406 Å) within the 2*θ* range from 10° to 80°. The Fourier transform infrared (FTIR) spectra were obtained through the application of an infrared spectrometer (PerkinElme, Spectrum RX I), utilizing the standard KBr disk methodology. Raman spectra were obtained using a Thermo Scientific Nicolet analyzer, using a laser excitation of 532 nm. Scanning electron microscopy (SEM) and energy-dispersive X-ray spectroscopy (EDS) were utilized to analyze the morphology and atomic ratio of content elements of the samples respectively (TSCAN, MIRA III). The nitrogen adsorption–depiction isotherms and the Brunauer–Emmett–Teller (BET) specific surface area of the powders were computed through the use of a surface area and pore size distribution analyzer (Belsorp, mini II). The photoluminescence (PL) spectra and ultraviolet-visible (UV-vis) transmission were acquired utilizing a UV-vis spectrometer (PHYSTECH, UVS-2500) and the excitation wavelengths for PL spectroscopy were 365 and 405 nm.

### 2.4. Photocatalytic test

**Fig. 2:**
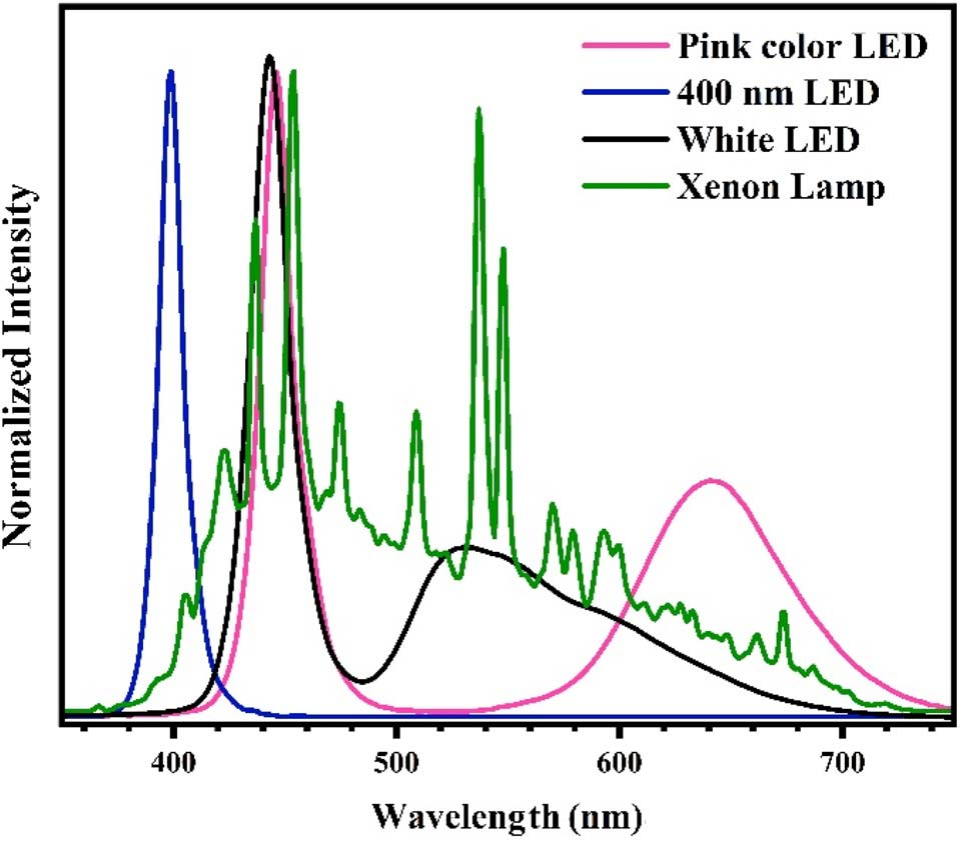
Optical spectra of light sources.

In order to evaluate the Cr(vi) photoreduction efficacy of photocatalysts produced at varied temperatures, a quantity of 20 mg of the powders underwent mixing with 15 mL of deionized water and subsequent ultrasound for 5 min. A volume of 5 mL of a citric acid solution with a concentration of 8 mM and 20 mL of a chromium(vi) solution with a concentration of 40 ppm was introduced into the colloidal photocatalyst. The mixture was subjected to stirring in dark for a period of 20 min to ensure attainment of the adsorption–desorption equilibrium, followed by exposure to a 55 W xenon lamp for a duration of 60 min. At intervals of 10 min, a 2 mL aliquot of colloid was taken and subjected to centrifugation for the purpose of photocatalyst separation. The concentration of Cr(vi) was evaluated utilizing the colorimetric technique employing diphenylcarbazide. Further experiments were conducted in a comparable fashion on the CN450 optimized sample, although with alterations made to the variables such as citric acid concentration with values of 0, 0.5, 1, 1.5 and 2 mM, pH with values of 2, 5, 8 and 11 and different light sources including white LED, 400 nm LED, pink color LED and xenon lamp which the spectra of light sources are displayed in Fig. 2. Furthermore, during the recycling test, the photocatalyst was retrieved through the use of a centrifuge and subjected to a rigorous rinse with deionized water. Citric acid and a freshly prepared solution of Cr(vi) in water were introduced into the colloid of the recycled sample.

## 3. Results and discussion

### 3.1. Structural properties and morphology

**Fig. 3:**
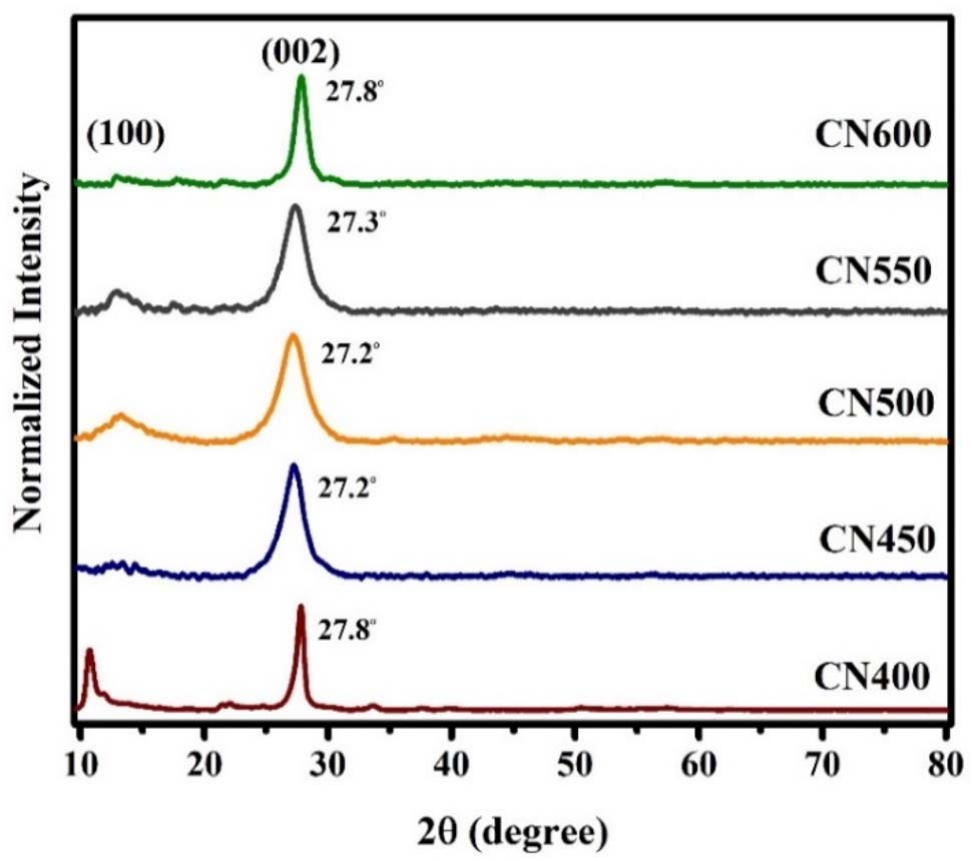
XRD patterns of g-C_3_N_4_ prepared at different temperatures.

XRD is a reliable methodology to validate and determine the crystal structure of graphitic carbon nitride. XRD patterns of carbon nitrides produced through varying calcination temperatures are depicted in Fig. 3 and consisting with the g-C_3_N_4_ JCPDS Card (No. 87-1526). The diffraction peak predominantly associated with the (002) plane is typically observed within the angular range of 2*θ* = 27.2°–27.8°, which arises due to the interlayer accumulation of the conjugated aromatic system and when the temperature got higher, it initially went down and then went back up. As the temperature of calcination is elevated, a gradual decline is observed in the diffraction angle, followed by an eventual increase. Similarly, there is an initial advancement in peak width, yet this subsequently gives way to a reduction. Furthermore, the aforementioned peak, which is more discernible in CN500 and CN550 samples at 13.1°, corresponds to the in-plane repetition of the tri-*s*-triazine unit. The XRD pattern obtained from the sample synthesized at a temperature of 400 °C exhibited a distinct peak at 11.2°, which is believed to correspond to intermediate compounds formed during the polymerization reaction of g-C_3_N_4_ at low temperature.^46^

**Fig. 4:**
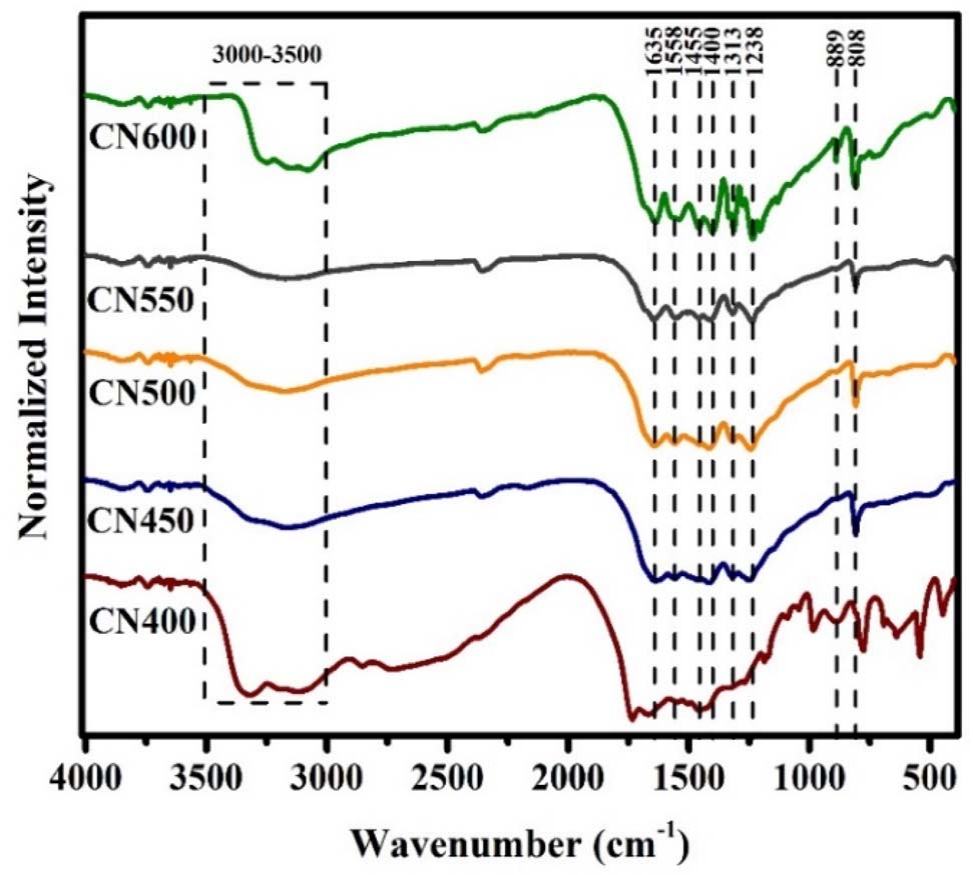
FT-IR spectra of g-C_3_N_4_ prepared at different temperatures.

The functional groups and chemical bonds contained in the produced g-C_3_N_4_ were investigated through exploration using FT-IR spectroscopy. The outcomes FT-IR spectra can be observed in Fig. 4. The samples exhibit analogous patterns within the spectrum spanning 1200–1650 cm^−1^, with a discernible increase in the sharpness of the absorption peak observable as the temperature of synthesis was heightened. The spectral peaks located at 808 and 889 cm^−1^ have been assigned to the breathing mode of tri-*s*-triazine and the deformation mode of N–H bonds, respectively.^47,48^ The peaks observed at around 1238, 1313, 1400, 1455, and 1558 cm^−1^ corresponded to the C–N bond and the peak at 1635 cm^−1^ was associated with C=N stretching vibration mode.^49–51^ In addition, a broad band in the range of 3000–3500 cm^−1^ indicates the existence of N–H stretching vibrations.^52^

**Fig. 5:**
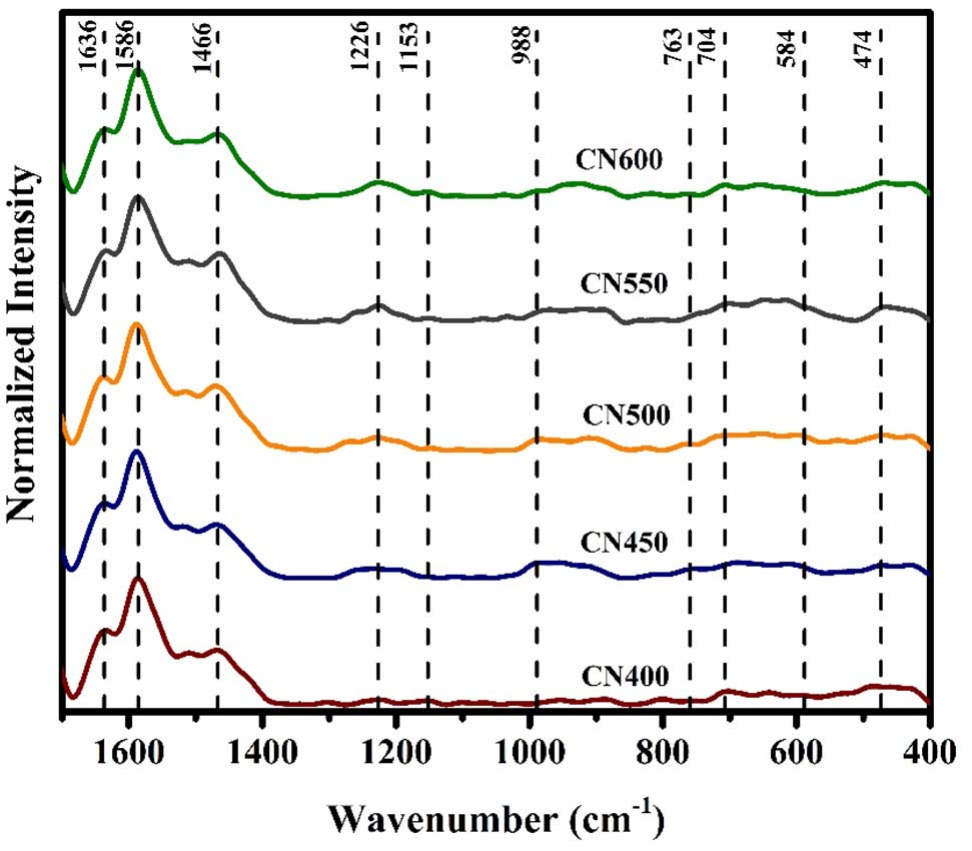
Raman shift spectra of g-C_3_N_4_ prepared at different temperatures.

The Raman spectra of prepared samples are depicted in Fig. 5. For g-C_3_N_4_, several characteristic peaks at 1636, 1586, 1466, 1226, 1153, 988, 763, 704, 584 and 474 cm^−1^ were identified, aligning to the typical vibration modes of CN heterocycles. Notably, the most intense peak in the g-C_3_N_4_ Raman spectra appears at 1586 cm^−1^ attributed to the ring-valence vibration of species *E*′. Additionally, the peak at 584 cm^−1^ in the lower frequency range is also associated with species *E*′, while the peak at 980 cm^−1^ corresponds to the breathing 1 mode of the triazine ring.^53,54^

**Fig. 6:**
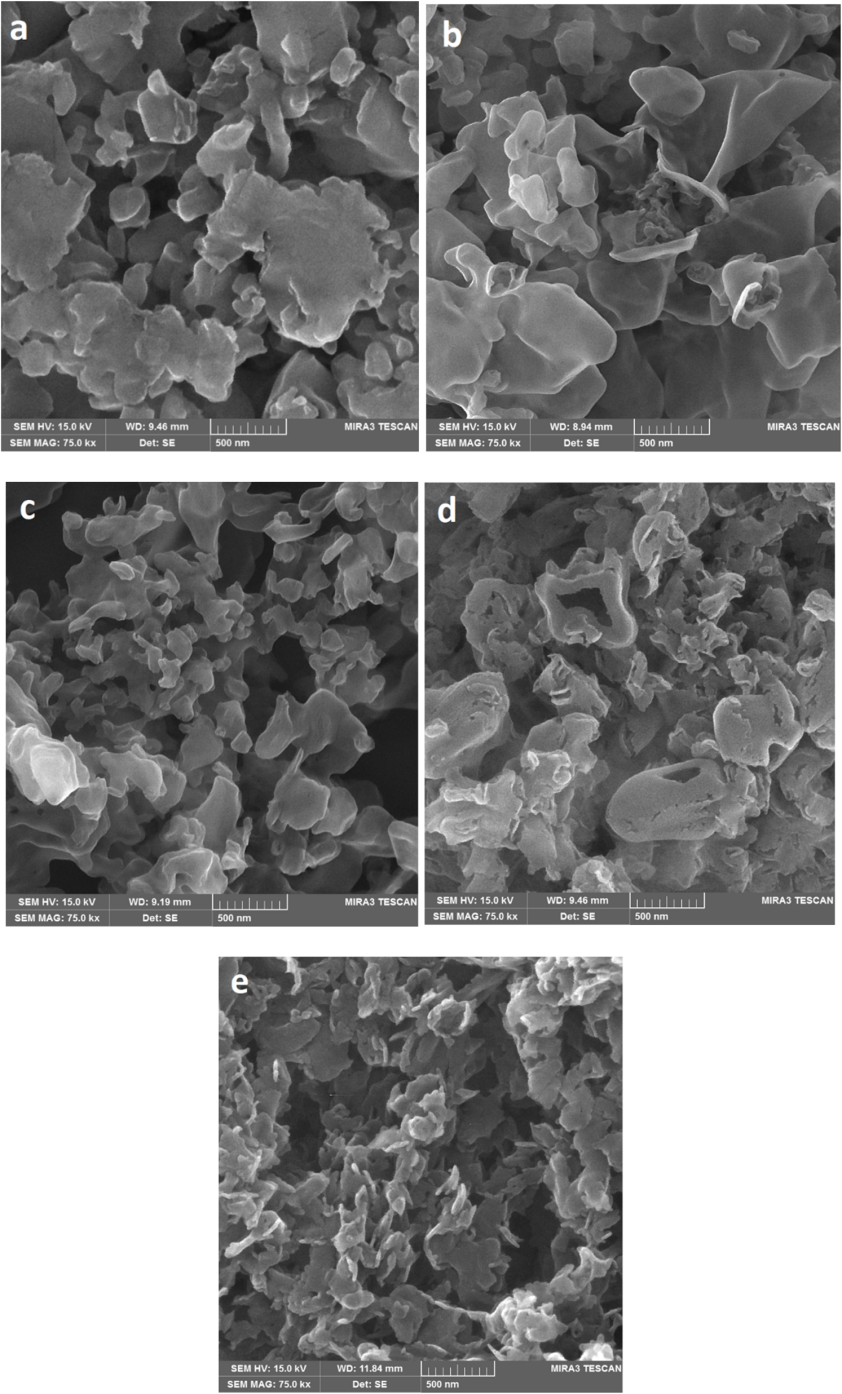
SEM images of (a) CN400, (b) CN450, (c) CN500, (d) CN550 and (e) CN600.

Fig. 6 depicts the SEM images acquired from the g-C_3_N_4_ samples. The images suggest that changes in the morphology are associated with a rise in temperature. The CN400 sample exhibits agglomerations of substances that are merged together. As the temperature approaches 450 °C, the morphology of the particles tends to exhibit a more plate-like geometry with increased intertwining.^27^ Upon reaching a temperature of 500 °C, smaller particles become evident, whereas exposure to 550 °C leads to the generation of cavities within smaller particles. Upon attaining a temperature of 600 °C, the particles underwent a transformation and assumed the morphology of small plates.^55^ The thickness of these particles was subsequently determined as 28 ± 1 nm. Also, the outcomes of the EDS analysis conducted on the powders, are presented in Table 1. The results reveal that the weight ratio of carbon to nitrogen elements exhibited an initial increase with rising temperature, followed by a subsequent decrease.

**Table 1:** Properties of prepared g-C_3_N_4_ synthesized at different temperatures

Sample	Product to urea mass ratio (%)	Band gap			BET		EDS atomic ratio (*W*%)		
		Tauc equation	PL (365 nm)	PL (405 nm)	*a*_s_ (m^2^ g^−1^)	Pore volume (cm^3^ g^−1^)	C	N	C/N
CN400	10.42	2.75	2.86	2.86	8.89	0.006	34.43	65.57	0.53
CN450	4.63	2.65	2.79	2.81	23.68	0.181	35.08	64.92	0.54
CN500	3.96	2.59	2.70	2.70	33.53	0.217	42.24	57.76	0.73
CN550	2.87	2.65	2.70	2.70	67.44	0.524	35.56	64.44	0.55
CN600	1.42	2.89	2.77	2.73	66.84	0.688	34.13	65.87	0.52

**Fig. 7:**
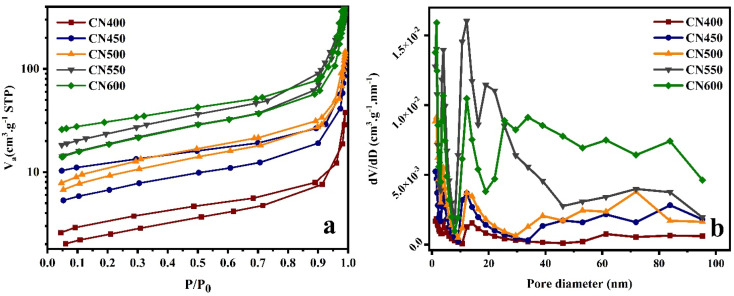
(a) N_2_ adsorption–desorption isotherms and (b) corresponding pore size distribution curves.

The surface area and porosity of the samples were assessed utilizing N_2_ adsorption–desorption isotherms. The findings reveal that the samples exhibit an IV adsorption–desorption isotherm displaying a H3 hysteresis loop, indicating the presence of a mesoporous configuration, as illustrated in Fig. 7. The surface area and pore volume of the analyzed specimens were computed and recorded in Table 1, presenting a comprehensive summary of the results. The observed phenomenon may be attributed to variations in the bubbling and oxidation of intermediate compounds that arise during the process of calcination. These outcomes appear to adhere to the morphology alterations that were witnessed during SEM. These factors ultimately contribute to a morphological transformation of the densely packed g-C_3_N_4_ to a porous configuration.^31,56^

### 3.2. Optical properties

**Fig. 8:**
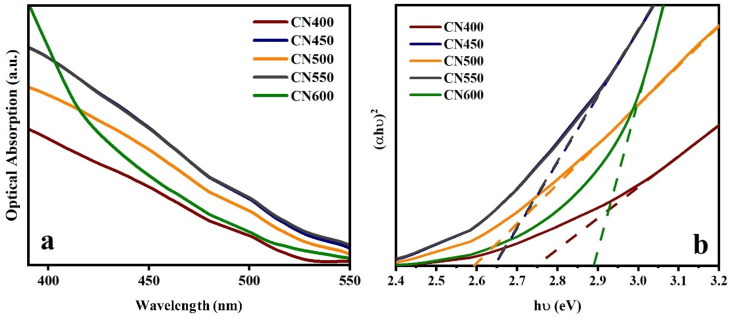
(a) UV-vis spectra and (b) Tauc equation plot of g-C_3_N_4_ synthesized at different temperatures.

Another important point to consider when evaluating the catalytic activity of a photocatalyst is its ability to respond to light which affects the band gap and light harvesting performance. The optical characteristics of the specimens were examined utilizing UV-vis spectroscopy, and the respective optical absorption spectra are depicted in Fig. 8a. The spectral analysis of CN600 exhibits a discernibly distinct pattern of light absorption in the range of 200 to 550 nm, in comparison to the absorption spectra displayed by other test samples. The absorption spectra of CN450, CN500, and CN550 exhibit comparability, and the spectra of CN450 and CN550 demonstrate a certain degree of similarity. The study conducted aimed to determine the band-gap energies of various samples that were synthesized at varying temperatures. The band-gap energies of samples synthesized at 400, 450, 500, 550, and 600 °C were calculated by the Tauc plot (*αhν*)^2^*versus hν*, and the estimated values were respectively 2.75, 2.65, 2.59, 2.65, and 2.89 eV as can be observed in Fig. 8b.

**Fig. 9:**
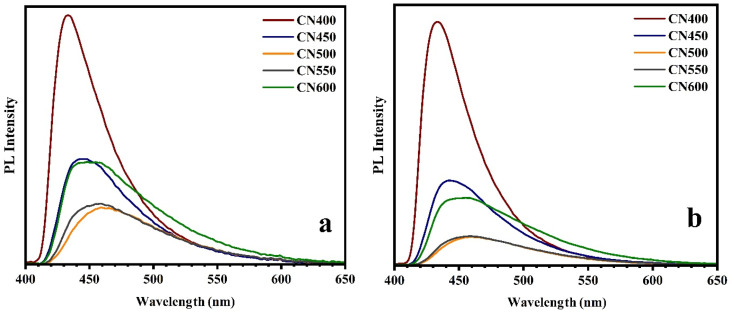
PL spectra of g-C_3_N_4_ synthesized at different temperatures by (a) 365 nm (b) 405 nm light source.

To improve the thoroughness of the analysis, photoluminescence (PL) spectra were employed to examine the optical properties of g-C_3_N_4_ powders using two distinct excitation light sources with wavelengths at 365 and 405 nm. The PL spectra are illustrated in Fig. 9a and b, for excitation wavelenghts at 365 and 405 nm respectively. The emission peaks observed at distinct wavelengths are attributable to the recombination of electron–hole, which releases light energy equivalent to the band gap energy of the samples of g-C_3_N_4_. The energy band gap values obtained through calculation of PL spectrum peaks are presented in Table 1.

### 3.3. Photocatalytic activity

**Fig. 10:**
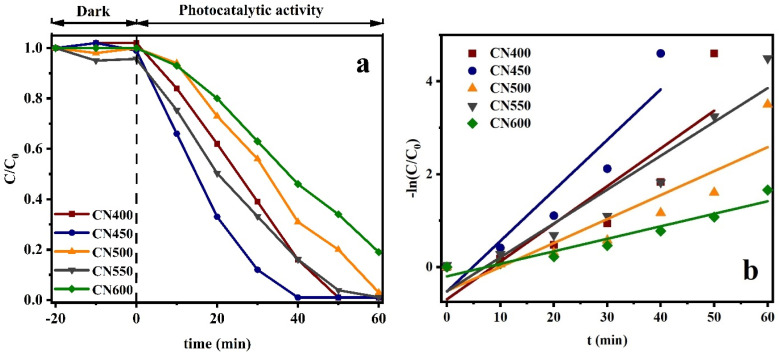
(a) Effect of calcination temperature on the photocatalytic activity of g-C_3_N_4_, (b) photocatalytic kinetic constants of g-C_3_N_4_.

The effectiveness of g-C_3_N_4_ samples, obtained at different calcination temperatures, was examined in the photoreduction of Cr(vi). As illustrated in Fig. 10a, the g-C_3_N_4_ photocatalysts demonstrate an insufficient capability to adsorb Cr(vi) potentially attributed to a feeble electrostatic attraction between the anionic chromate species and the negatively charged surface of the catalyst.^37^ During the period of exposure to radiation, the samples exhibited different degrees of photocatalytic performance. CN450 displayed the highest level of proficiency in reducing Cr(vi) with an efficiency rate of over 99% in just 40 min, compared to CN400 which achieved the same level of efficiency within 50 min. The results indicate that a minimum duration of one hour is necessary for CN500 and CN550 to exhibit a photoreduction rate of more than 99%. Additionally, CN600 demonstrates an 80% photoreduction rate during the identical time duration. The pseudo-first-order method was used to determine the kinetics of chromium photoreduction. Eqn (1) was utilized, where *C*_0_, *C*, *k* and *t* are concentrations of Cr(vi) in solution at time 0, concentrations of Cr(vi) in solution at time *t*, rate constant and reaction time respectively. Also, a visual representation of the results can be seen in Fig. 10b.1
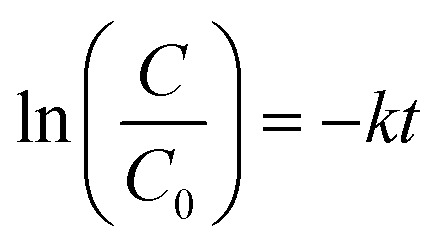


The values of (*k*) for samples CN400, CN450, CN500, CN550 and CN600 were calculated, respectively, 0.0813 min^−1^, 0.1089 min^−1^, 0.0517 min^−1^, 0.0729 min^−1^, and 0.0270 min^−1^.

**Fig. 11:**
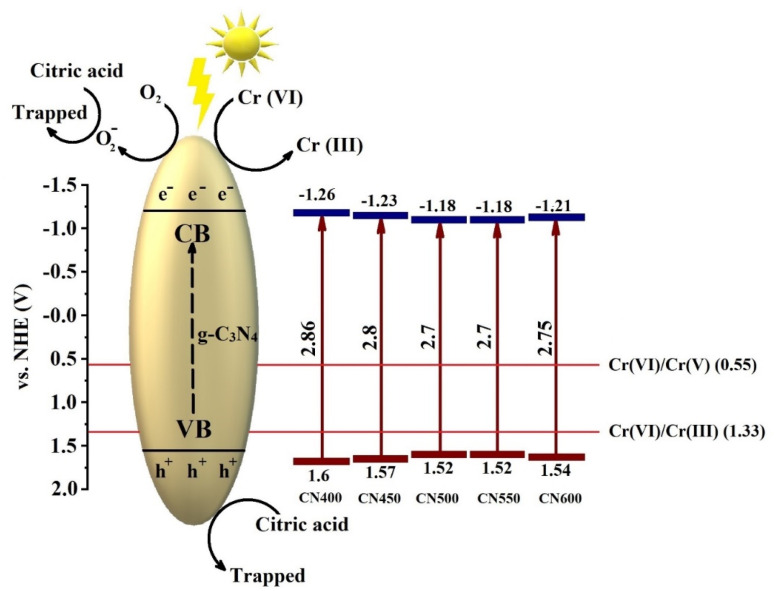
Band position and Cr(vi) photoreduction mechanism of g-C_3_N_4_.

To calculate the position of band structure of g-C_3_N_4_ and explore the mechanism of photoreduction of Cr(vi) eqn (2) and (3) were utilized. where *E*_CB_ and *E*_VB_ represent the conduction band (CB) and valence band (VB) edge potentials, respectively, *E*^e^ (∼4.5 eV) is the free electron energy on the scale of hydrogen, *X* represents the electronegativity of the semiconductor and *E*_g_ is the semiconductor band gap. The value of *X* for g-C_3_N_4_ was calculated as 4.67 eV and by referring eqn (2) and (3) the values of calculated VBs and CBs were shown in Fig. 11.^57^ Moreover, chromium metal is present in two stable oxidation states, Cr(vi) and Cr(iii).^58^ Given that the colorimetric diphenylcarbazide method exclusively identifies Cr(vi), it is anticipated that the quantity of reduced Cr(vi) has converted to Cr(iii).^59^ Based on the calculated values of CBs and VBs, a possible mechanism of photocatalytic Cr(vi) reduction within the presence of citric acid has been suggested as appeared in Fig. 11. The photogenerated electrons in the CB of g-C_3_N_4_ are transferred for the photocatalytic reduction of Cr(vi) to Cr(iii), and at the same time the citric acid which acts as a sacrificial agent is trapped by photogenerated holes in the VB of g-C_3_N_4_.2
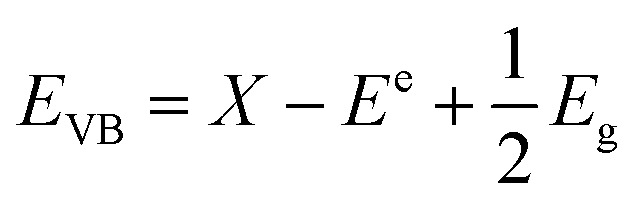

3 *E*_CB_ = *E*_VB_ − *E*_g_

**Fig. 12:**
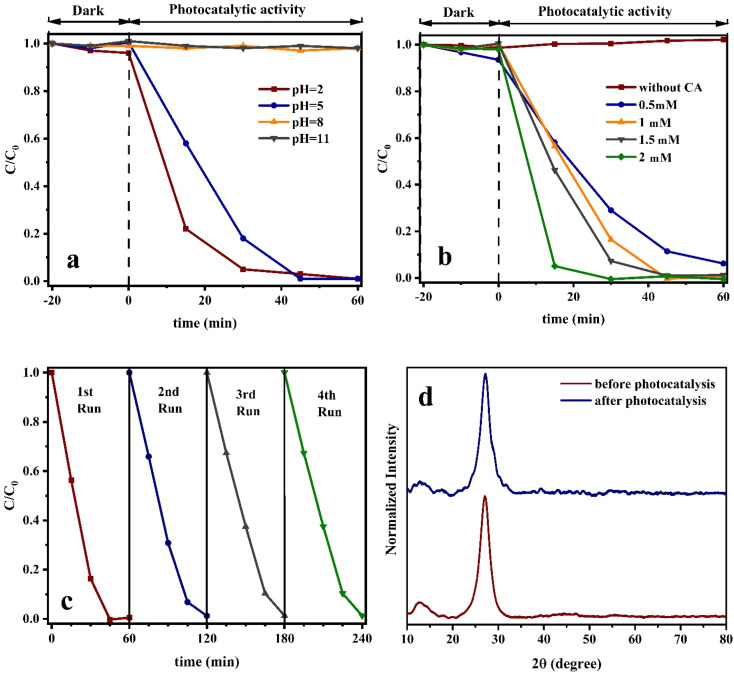
(a) Solution pH, (b) citric acid concentration, (c) recycling effects on CN450 photoreduction of Cr(vi) process under xenon lamp and (d) XRD patterns of CN450 before and after photocatalysis.

The effect of other process variables on g-C_3_N_4_ photocatalytic performance such as pH, citric acid concentration, and recycling ability was investigated under xenon lamp on CN450 and the results are shown in Fig. 12a–c respectively. The effectiveness of the photocatalytic reduction process for Cr(vi) is observed to decrease in a substantial manner with an increase in the pH value as mentioned in Fig. 12a. In addition, when pH is 8 and 11, Cr(vi) cannot be reduced at all. In general, the Cr(vi) species are commonly found in tetrahedral oxo-compounds, specifically in the form of CrO_4_^2−^, at pH levels greater than 5.0, and while they appear as Cr_2_O_7_^2−^ or HCrO_4_^−^ at neutral or acidic pH levels ranging from 2.0 to 5.0. Furthermore, the surface charge of the photocatalyst, attributed to the pH level, constitutes a factor that partially accounts for the observed phenomenon.^37,60^ The results indicate that an excess quantity of hydrogen ions (H^+^) is present, namely, a state of acidosis. The phenomenon of reduction from photogenerated electrons is stimulated in an environment with increased acidity, as depicted through eqn (4) and (5):^37^

4 Cr_2_O_7_^2−^ + 14H^+^ + 6e^−^ → 2Cr^3+^ + 7H_2_O

5 HCrO_4_^−^ + 7H^+^ +3e^−^ → Cr^3+^ + 4H_2_O

In alkaline conditions, electrostatic repulsion between g-C_3_N_4_ and Cr(vi) impeded the interaction with photogenerated electrons, resulting in a decrease in the Cr(vi) removal rate by means of eqn (6):^37^

6 CrO_4_^2−^ + 4H_2_O + 3e^−^ → Cr(OH)_3_ + 5OH^−^

According to active species trapping experiments especially, *tert*-butyl alcohol, 2,2,6,6-tetramethylpiperidine-1-oxyl, and methanol scavengers, the efficacy of Cr(vi) removal in photocatalytic reduction reactions mediated by g-C_3_N_4_ can be enhanced through the augmentation of organic acids acting as hole-scavenging agents. Photoreduction is the mechanism through which Cr(vi) is reduced in water, with the electrons produced in the CB through photon absorption playing a crucial role in the process. The catalytic process of water oxidation leading to the creation of molecular oxygen is primarily driven by the photogenerated holes found in the valence band. As water oxidation is a complex process that determines the rate and it involves a four-electron transfer reaction. Thus, to attain optimal reduction of Cr(vi) while separating electron–hole pairs efficiently, it is necessary to use a hole scavenger. Therefore, the augmentation of the chromium reduction rate as illustrated in Fig. 12b can be rationalized by the amplification of the citric acid concentration.^61–63^ It should be noted, citric acid monohydrate without any semiconductor can provide Cr(vi) photoreduction under 400 W metal halide lamp as a light source, therefore should be aware about the role of citric acid in Cr(vi) photoreduction. However, it should be mentioned the best performance of citric acid monohydrate Cr(vi) photoreduction observed at higher concentration under more than 300 W metal halide and high-pressure mercury lamps.^64^

Fig. 12c exhibits the recuperation rate of the CN450 photocatalyst subsequent to each cycle of the photocatalytic procedure for the purpose of Cr(vi) reduction. Notwithstanding a decrease in the photocatalyst quantity during successive rounds, the fourth round demonstrated considerable efficacy in reducing the concentration of chromium to above 98% of its initial level. Additionally, XRD analysis was conducted following four cycles of photoreduction, as illustrated in Fig. 12d. The CN450 material exhibited successful reutilization, showcasing an almost stable photocatalytic performance.

**Fig. 13:**
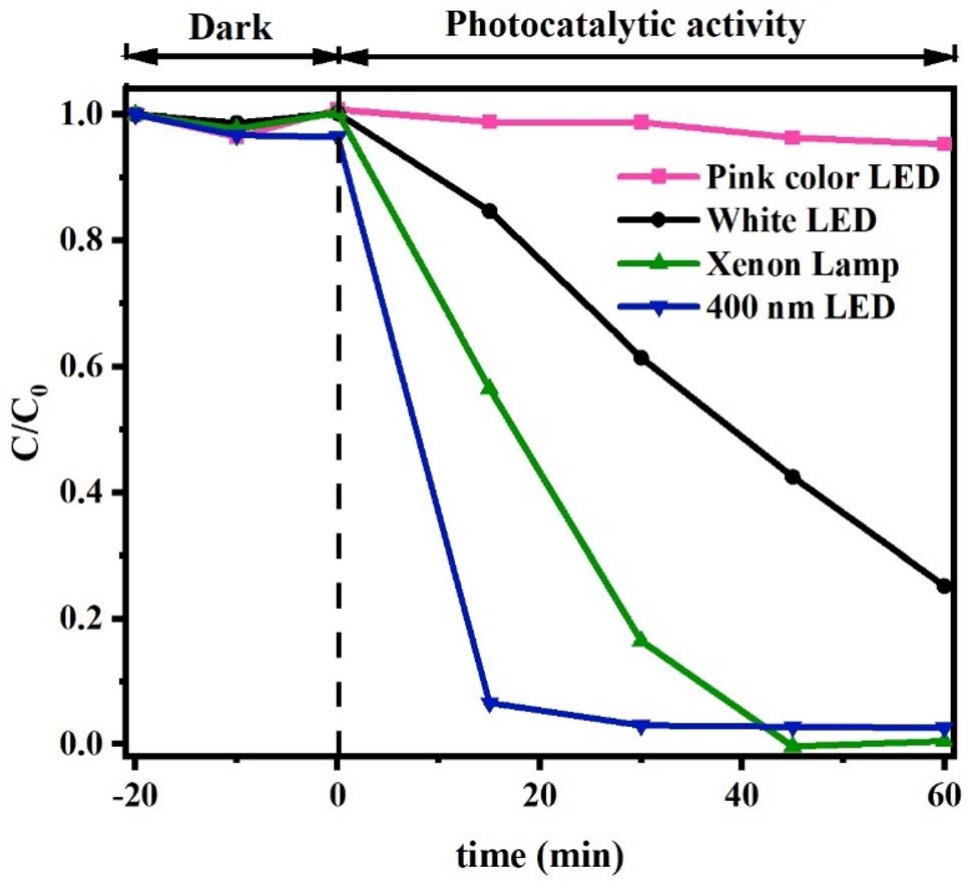
Light sources effects on CN450 photoreduction of Cr(vi) process.

Photocatalytic materials encounter another challenge in their activation by light, as it hinges upon the spectral composition of the incident light and the bandgap energy of the photocatalyst, necessitating optimal alignment between the two. The use of solar radiation has been widely recognized as a highly advantageous approach to activating the photocatalyst, owing to its environmentally friendly nature. Consequently, due to the comparable and full range of the visible spectrum emitted by the xenon lamp, it has been frequently utilized at high power of intensity in numerous photocatalytic research. In contrast, scholars have investigated the viability of visible LED as source. This approach stems from the fact that the intensity of sunlight is contingent upon the geographical position and time of day, however, the conversion of electrical energy into light *via* xenon lamps leads to a notable production of heat, the efficacy of LED lamps has also undergone comprehensive examination. Such an investigation is elucidated in Fig. 13. As hypothesized, the efficiency of LED lamps is observed to increase in proportion to the reduction of their radiation wavelengths. The present findings indicate a heightened photocatalytic efficiency in the 400 nm LED light source, which exhibits a lower power consumption compared to its xenon-based counterpart.

## 4. Conclusion

In this research, structural and optical properties and also photocatalytic activity of g-C_3_N_4_ synthesized by different calcination temperatures were investigated. The results of investigation revealed that the morphology and structure of g-C_3_N_4_, as well as its photocatalytic capability in reducing Cr(vi), are considerably influenced by the synthesis calcination temperature. Additionally, the findings have demonstrated that elevating the synthesis temperature results in a substantial augmentation of the specific surface area of product. Nonetheless, it is imperative to acknowledge that the augmentation of the specific surface area is not always indicative of enhanced outcomes in the context of g-C_3_N_4_ photocatalysis. Also, the calcination temperatures, the concentration of citric acid as a hole scavenger, the source of brightening, pH levels, and the reusing capacity of the sample with highest efficiency were examined. When there is more citric acid and the pH level goes down, the photocatalyst works better. Among the four cases we looked at for the light source, we found that the 400 nm LED worked the best. Furthermore, even after four times going through the process of reducing under light, the photocatalysis reaction has still remained highly efficient.

## Data availability

The data that support the findings of this study are available from the corresponding author upon request.

## Conflicts of interest

There are no conflicts to declare.
